# Sensory neuron transcriptomes reveal complex neuron-specific function and regulation of *mec-2/*Stomatin splicing

**DOI:** 10.1093/nar/gkab1134

**Published:** 2021-12-08

**Authors:** Xiaoyu Liang, Canyon Calovich-Benne, Adam Norris

**Affiliations:** Southern Methodist University, Dallas, TX 75275, USA; Southern Methodist University, Dallas, TX 75275, USA; Southern Methodist University, Dallas, TX 75275, USA

## Abstract

The function and identity of a cell is shaped by transcription factors controlling transcriptional networks, and further shaped by RNA binding proteins controlling post-transcriptional networks. To overcome limitations inherent to analysis of sparse single-cell post-transcriptional data, we leverage the invariant *Caenorhabditis elegans* cell lineage, isolating thousands of identical neuron types from thousands of isogenic individuals. The resulting deep transcriptomes facilitate splicing network analysis due to increased sequencing depth and uniformity. We focus on mechanosensory touch-neuron splicing regulated by MEC-8/RBPMS. We identify a small MEC-8-regulated network, where MEC-8 establishes touch-neuron isoforms differing from default isoforms found in other cells. MEC-8 establishes the canonical long *mec-2/Stomatin* isoform in touch neurons, but surprisingly the non-canonical short isoform predominates in other neurons, including olfactory neurons, and *mec-2* is required for olfaction. Forced endogenous isoform-specific expression reveals that the short isoform functions in olfaction but not mechanosensation. The long isoform is functional in both processes. Remarkably, restoring the long isoform completely rescues *mec-8* mutant mechanosensation, indicating a single MEC-8 touch-neuron target is phenotypically relevant. Within the long isoform we identify a cassette exon further diversifying *mec-2* into long/extra-long isoforms. Neither is sufficient for mechanosensation. Both are simultaneously required, likely functioning as heteromers to mediate mechanosensation.

## Graphical Abstract

**Graphical Abstract:**
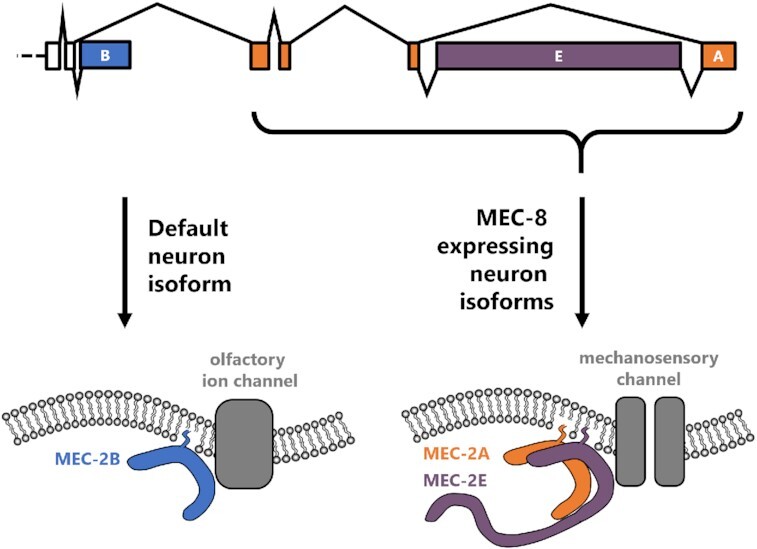
mec-2 isoforms have distinct expression, regulation and function in specific sensory neurons.

## INTRODUCTION

The function and identity of a single cell is shaped by the complement of genes it expresses. Recent advances in single-cell transcriptomics have enhanced our understanding of how gene expression is regulated at the level of individual cells (1). In the nervous system, for example, single-cell RNA-seq has revealed new cellular sub-types and subtype-specific transcription factors within the mammalian brain (2,3).

The function and identity of a single cell can also be shaped by post-transcriptional regulation of gene expression. For example, functional neuronal classes can be defined by specific alternative splicing isoforms or the expression of specific RNA binding proteins (4,5). In contrast with single cell RNA-seq for gene expression analysis, measuring isoform expression in single cells is technically challenging due to low sequencing depth and uniformity of coverage (6,7). These limitations can lead to erroneous conclusions. For example, sparse coverage can lead to the incorrect conclusion of binary splicing choices in which only one isoform of a given gene is expressed per cell (7). We therefore know less about how splicing regulation contributes to the identity and function of single neurons.

Given an individual single-cell transcriptome, it is often unclear (i) what are the regulatory mechanisms by which it is generated, and (ii) which elements of the regulatory network are functionally relevant? A related challenge is, given a network of genes or isoforms controlled by a regulatory protein, which are the functionally-relevant targets? For example, a number of RNA binding proteins have been implicated in human neuronal disorders, but even when the relevant neuronal cell type can be identified, it often remains a mystery as to which target(s) contribute to the phenotype (8). This speaks to the challenge of extracting regulatory and functional information from single-cell gene regulatory networks.

We set out to address these challenges in the context of the nervous system of the nematode *Caenorhabditis elegans*. To address the regulation of alternative splicing networks in single cell types, we took advantage of the invariant *C. elegans* cell lineage. In *C. elegans* a single cell type can be genetically labeled in thousands of individual isogenic animals, then the animals can be dissociated and thousands of the same cell type can be collected and subjected to sequencing (9). The resulting high sequencing depth and uniformity facilitates the analysis of splicing regulatory networks in single cell types.

To address the question of functionally-relevant targets within splicing networks, we focused on a well-described neuronal cell type responsible for a straightforward and quantitative behavior. The mechanosensory ‘touch neurons’ respond to gentle mechanical stimuli delivered to the worm's body. Touch neurons can be labeled with specific GFP reporters and their function can be assessed by measuring the animal's avoidance response to gentle touch administered by an eyelash hair. Classic genetic screens identified the conserved splicing regulator MEC-8/RBPMS as a gene essential for touch neuron function (10,11). Careful examination of the MEC-8-regulated transcriptome of touch neurons may therefore reveal regulatory and functional insights into splicing networks in specific neuron types.

Using cell specific RNA-seq of wild-type and *mec-8* mutant touch neurons, we identify a small network of 13 MEC-8-regulated isoforms, compared to dozens of isoforms regulated by MEC-8 in whole animals. MEC-8 establishes a number of touch neuron-specific isoforms distinct from the ‘default’ splicing pattern found in other cell types. For example, many neurons express a non-canonical short isoform of the conserved mechanosensory gene *mec-2/Stomatin*, while MEC-8 mediates expression of a long isoform in touch neurons. This observation leads to the identification of a role for *mec-2* in olfaction, in addition to its well-known role in mechanosensation. The short isoform functions in olfaction but not in touch sensation, while the long isoform functions in both. Remarkably, *mec-8* mutant touch sensation is fully rescued by genetic restoration of a single MEC-8 target, the *mec-2* long isoform. Finally, within the long *mec-2* isoform we identify additional splicing diversity: a previously-uncharacterized large alternative exon yields either an extra-long *mec-2* isoform when included, or the canonical long *mec-2* isoform when skipped. Both isoforms are expressed in touch neurons and are simultaneously required for touch sensation, likely as heteromultimers. In sum, by analyzing deep cell-specific transcriptomes, we uncover both regulatory and functional aspects of alternative splicing in a single neuron type, with implications for the function of different types of sensory neurons.

## MATERIALS AND METHODS

### *C. elegans* strain maintenance and generation

*Caenorhabditis elegans* were maintained by standard techniques on NGM agar plates seeded with OP50 *Escherichia coli* at 20°C. Reference alleles *mec-8(e398)* and *mec-2(e75)* used unless otherwise noted. Transgenic animals bearing extrachromosomal arrays were injected at 20 ng/μl. CRISPR/Cas9 transgenics were generated using dual plasmid gRNAs and plasmid homologous repair templates. All mutant lines were verified for seamless editing using Sanger sequencing and subsequently outcrossed. Double edits were performed sequentially, with the first edit homozygosed and sequence verified before proceeding to the second edit.

Strains generated in this study: ADN301 = Ex[MEC-8::GFP fosmid]. ADN1057 = *mec-8(e398);* Is[*mec-4p::GFP*]. ADN959 = Ex[*mec-3p::elp-1* exon 5 splicing reporter]. ADN960 = Ex[*rgef-1p::elp-1* exon 5 splicing reporter]. ADN999 = *mec-8(csb22);* Ex[*mec-3p::elp-1* exon 5 splicing reporter]. ADN1000 = *mec-8(smu1)*; Ex[*rgef-1p::elp-1* exon 5 splicing reporter]. PHX3660 = *elp-1(syb3660)*. PHX2112 *= mec-2(syb2112)* MEC-2A*::wrmScarlet*. PHX4210 = MEC-2A::wrmScarlet MEC-2E::3XFlag. PHX3795 = *mec-2e(syb3795)* = MEC-2E::wrmScarlet. PHX1764 = *mec-2(syb1764) mec-2B* isoform forced expression. ADN758 = *mec-2A* isoform forced expression. ADN759 = *mec-2E* isoform forced expression. ADN446 = Ex[MEC-2B::GFP fosmid]. PHX strains were custom ordered and generated by SunyBiotech. We previously generated *mec-8* deletion allele *csb22* and demonstrated it to be a null mutant (12). Additional strains were provided by the CGC, which is funded by NIH Office of Research Infrastructure Programs (P40 OD010440).

### Behavioral analysis

For gentle touch assays, an eyelash hair was used to gently stroke either the anterior body (just posterior to head) or posterior body (just anterior to anus). Total of 10 strokes were performed on each worm (five to anterior body, five to posterior body). Worms usually moved backwards or stopped moving forward when gentle touch was performed to anterior body, while worms moved forward quickly when gentle touch was performed to posterior body. Avoidance of touch was considered as positive response, and a total number of positive response (out of 10) was recorded. At least 10 worms were used for gentle touch assay.

Olfaction assays were performed as previously described (13). Unseeded plate were divided into four quadrants, two with buffer and two with odorant, chemotaxis index = (# animals in two odorant quadrants)/(# animals in any of the four quadrants) The following modifications were made: animals were staged at larval L4 stage, M9 buffer was used for washes, and animals were pelleted via gravity rather than centrifugation to minimize bacterial co-transfer, since *mec-2* loss-of-function mutants are lethargic in the presence of even trace amounts of bacterial food.

### Cell dissociation and cell sorting

Worms were grown on 100 mm NGM agar plates seeded with thick OP50 lawn. To obtain synchronized L4 worms, embryos were isolated from gravid hermaphrodites using hypochlorite solution then hatched overnight in M9 buffer at room temperature. Next day, synchronized L1 worms were put onto OP50 seeded plates for 36–40 hours at room temperature. L4 stage was verified by vulva morphology.

Dissociated cells were prepared as previously reported (9,14) with minor modifications. Briefly, L4 worms were washed with M9 buffer twice to remove bacteria. Worm pellet (∼400 μl) then incubated with 800 μl of SDS-DTT solution (200 mM DTT, 0.25% SDS, 20 mM HEPES, 3% sucrose, pH 8.0) to disrupt cuticle for 4 min. After SDS-DTT treatment, worms were washed with 1× egg buffer for five times. Worms then incubated with 20 mg/mL pronase (dissolved in 1 x egg buffer, Sigma-Aldrich P8811) from *Streptomyces griseus* for 30 minutes. During incubation, worms were disrupted by pipetting for 80 times per 5 min. When most of worms were dissociated, 1× egg buffer was added to stop the digestion and cells pelleted by centrifuging at 1500 rcf for 5 min at 4°C. The pellet was resuspended in 1× egg buffer, washed a second time to remove pronase completely. Dissociated cells were separated from worm debris by centrifuging 100 rcf for 2 min at 4°C. Supernatant which contains cells was filtered using 40-μm filter into collection tube.

Sorting experiments were performed on SONY SH800S cell sorter equipped with a 70-μm diameter chip. N2 strain (non-GFP cells) was used to set up appropriate gates for *mec-4p::*GFP strains (zdIs5, GFP-expressing touch receptor neurons) to exclude auto-fluorescent cells. GFP+ cells and corresponding GFP- cells were sorted into 500 μl TRIzol respectively for RNA extraction.

### RNA extraction, library preparation, sequencing and analysis

RNA was extracted with Direct-zol RNA Miniprep Kits (Zymo research R2050) following the protocol. DNA libraries were prepared for sequencing using the NEBNext Single Cell/Low Input RNA Library Prep Kit for Illumina according to standard manufacture's protocol. Paired-end 150 bp sequencing mapped with STAR (15) (version 2.5.3) using *C.elegans* reference genome WBcel235 yielded 35–100 million uniquely mapped reads for each sample. Parameters used in STAR are as follows: – outFilterMultimapNmax10–alignSJoverhangMin5–alignSJDBoverhangMin3–outFilterMismatchNmax10. The sequencing data generated in this study are available at the NCBI SRA archive (PRJNA752629) and GEO (GSE184149).

Alternative splicing was analyzed using JUM (16) (version 2.0.2) requiring exon junctions to be represented by at least ten junction-spanning reads in at least two out of three replicates to be considered *bona fide* splicing events. Significance values were set at FDR-corrected *q* < 0.05 with a Percent Spliced In |ΔPSI| of >15%. JUM reports global alternative splicing patterns, defines, and assembles them into conventionally known patterns: alternative 5′ splice site; alternative 3′ splice site; cassette exon; intron retention; mutually exclusive exon, as well as composite (an additional previously unclassified alternative splicing pattern, which is composed of different conventionally recognized alternative splicing patterns). Quantification of changes in splicing patterns between different conditions (e.g. wild-type and *mec-8* mutant touch neurons) were represented as percent spliced in values (ΔPSI). A False Discovery Rate (FDR)-adjusted *P* values (or *q* value) <0.05 were considered statistically significant. Differential expression analysis between different conditions was performed by DESeq2 (17) (version 1.26.0) with default settings. Gene expression enrichment in touch neurons and *mec-8*-regulated splicing events can be found in the Supplementary Spreadsheet. For prediction of intrinsically-disordered domains, we used PrDOS (18) under default parameters, including 5% false positive rate.

### Microscopy

Images of *elp-1* splicing reporter and *mec-2* endogenously-tagged strains were obtained with a Zeiss AxioImager Z1 and processed in ImageJ. For immunofluorescence of mec-2A::RFP + mec-2E::FLAG Immunohistochemistry, large pellets of gravid animals were collected, and the following protocol was followed: fresh fixing reagent: 400 μl MeOH, 100 μl Molecular water, 300 μl 8% PFA, 20 μl βME. animal pellet was flash frozen in liquid nitrogen after rotating pellet in fixation reagent. Worms were cracked using hot water bath and flash freezing in liquid nitrogen, washed four times with triton–borate buffer, five times with βME-triton-borate buffer, five times with PBST until βME smell dissipated. Primary antibody was added at 1:300 or 1:400 depending on affinity of antibody, incubated overnight at 4°C, washed five times with PBST. Secondary antibody was added at 1:400 and incubated overnight at 4°C, washed five times with PBST. 20 μl stained animal pellet was mounted to slide, and anti-glare media added to animal pellet and mixed on slide. Coverslip was added then sealed coverslip with clear nail polish.

## RESULTS

### Cell-specific sequencing of wild-type and *mec-8* mutant touch neuron transcriptomes

**Figure 1.**
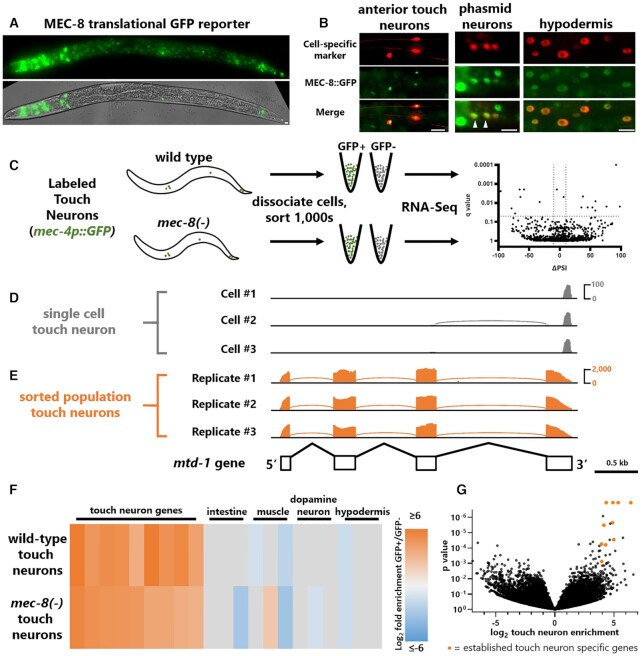
cell-specific sorting of wild-type and *mec-8* mutant touch neurons. (**A**) MEC-8 expression assessed by translationally-tagged transgenic fosmid reporter. Fluorescence is present in many tissues and cells throughout the worm. Scale bar represents 10 μm. (**B**) Translational fosmid reporter crossed with cell-specific RFP transgenes: *mec-17p* for anterior touch neurons, *osm-6p* for phasmid neurons (arrowheads indicate phasmid neurons, additional neuron is PQR), *dpy-7p* driving HIS-58::RFP for hypodermal nuclei. Scale bar represents 10 μm. (**C**) Schematic for obtaining sorted touch neurons and paired sorted negative control cells for both wild-type and *mec-8(-)* animals. Reference allele *mec-8(e398)* is used unless otherwise specified. (D, E) Re-analysis of existing touch neuron scRNA-seq from Taylor *et al.*, 2020 (**D**) in comparison with our touch neuron population RNA-seq (**E**) for the gene *mtd-1*, which is the highest-enriched gene from the scRNA-seq touch neuron data, highlighting the increase in coverage and splice junction representation afforded by sorted neuron sequencing. (**F**) Log_2_ fold changes determined by DESeq2 comparing GFP+ versus GFP- sorted cells for selected genes with literature-attested expression in specific cells or tissues. See also Supplementary Figure S1B for individual gene names. (**G**) Volcano plot of GFP+ versus GFP- enrichment for all detectable genes as determined by DESeq2, pooling both wild-type and *mec-8(-)* samples. Orange data points indicate genes known to be specific to (or highly enriched in) touch neurons based on existing single-gene analyses (in order of *P*-value magnitude: *mtd-1, mec-17, mec-4, mec-10, mec-18, mec-1, mec-7, mec-3, mec-12, mec-14*).

The conserved splicing factor MEC-8/RBPMS was first identified in *C. elegans* as a gene required for the function of mechanosensory ‘touch neurons’ (10,11). MEC-8/RBPMS homologues in vertebrates and invertebrates are expressed with both tissue specificity (*e.g*. vascular smooth muscle) and neuron-type specificity (*e.g*. retinal ganglion cells) (19,20). Likewise, *C. elegans* MEC-8 is expressed in specific tissues such as the hypodermis, as well as specific subsets of neurons (Figure 1A-B), notably the touch neurons and phasmid neurons (21,22) (Figure 1B).

To focus on the functional and regulatory activity of MEC-8 specific to touch neurons, we turned to cell-specific transcriptomics using established cell sorting approaches (9). We used a *mec-4p::GFP* transgene which labels all six touch neurons (ALML/R, AVM, PVM, PLML/R) in both wild-type and *mec-8* mutant animals (Figure 1C). Large numbers of synchronized L4-stage animals (from approximately 30 large 100 mm plates) were dissociated, and GFP-positive cells were sorted and sequenced in parallel with dissociated GFP-negative-sorted controls (Figure 1C). Experiments were performed in biological triplicate, yielding an average of 13,032 sorted cells per biological replicate, and polyA-selected RNA-seq yielded an average of 26,886,700 uniquely mapped reads per sample (Supplementary Figure S1A).

In contrast with single cell RNA-seq approaches designed to yield 3′ end-enriched reads for the purpose of gene expression analysis (1), our sorted neuron RNA-seq approach yields libraries with increased depth and uniformity of coverage throughout the gene body, thus enabling the analysis of splicing in single neuron types (Figure 1D, E). We observe strong enrichment for known touch neuron-specific genes in both wild-type and *mec-8* mutant sorted neuron populations (23), but not for genes specific to other tissues or neuronal cell types such as hypodermis or dopaminergic neurons (Figure 1F, G).

### MEC-8 establishes unique touch-neuron specific isoforms

**Figure 2.**
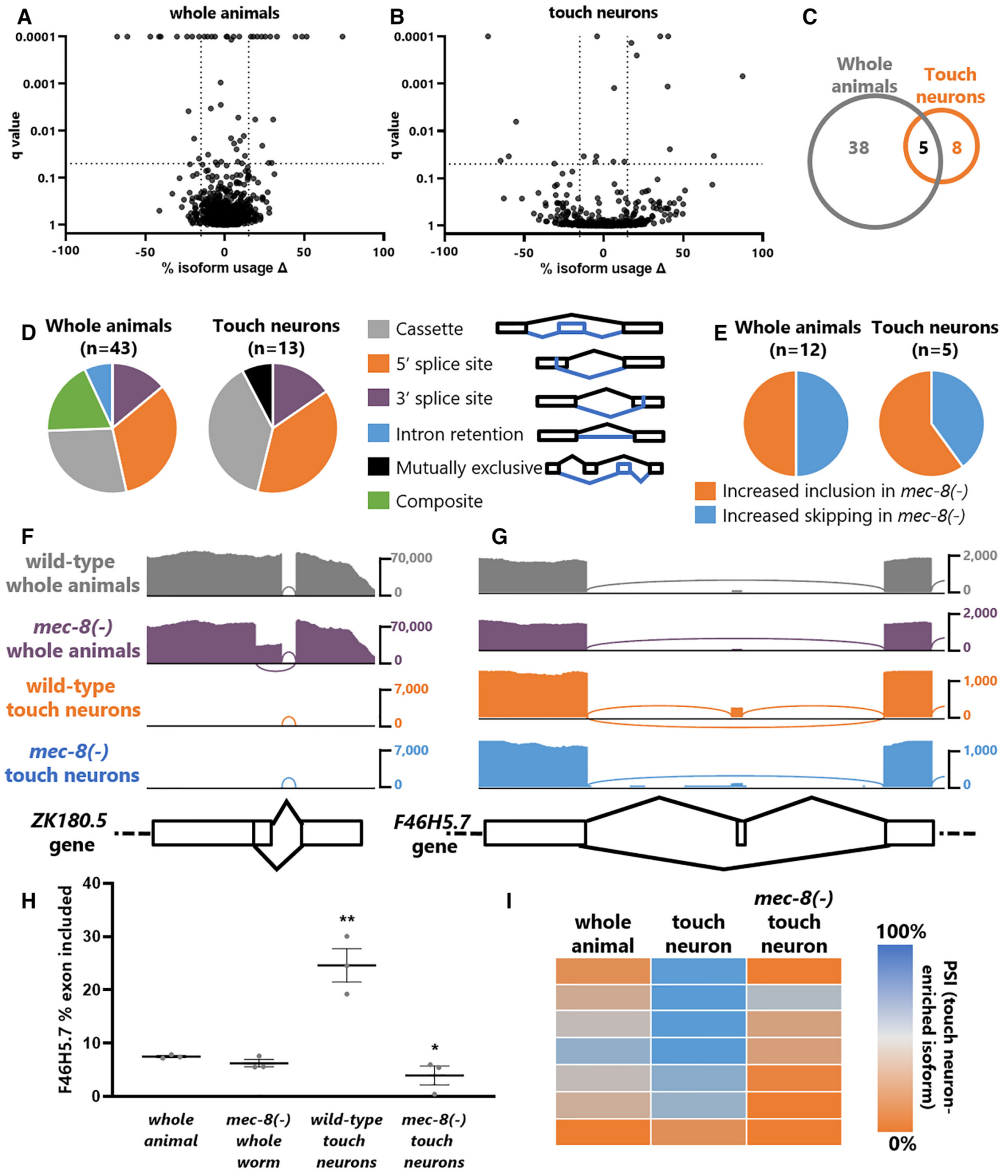
MEC-8-regulated isoforms in whole animals and in touch neurons. (A, B) Volcano plots representing change in isoform usage between wild-type and *mec-8(-)* mutants in populations of either undissociated whole animals (**A**) or dissociated sorted touch neurons (**B**), as assessed by JUM. Cutoffs drawn at |ΔPSI| > 15% and *q* value < 0.05. For alternative 5′ and 3′ splice site choices, the proximal splice site is arbitrarily defined as a positive value. (**C**) Number of alternative splicing events between wild-type and *mec-8(–)* detected in both touch neuron and whole animal comparisons, versus those detected in only one analysis, |ΔPSI| > 15% and *q* value < 0.05. (**D**) Types of MEC-8-regulated alternative splicing detected in whole animals and in touch neurons. ‘Composite’ refers to a complex mixture of splicing events that cannot be reduced to simple constituent splicing choices. (**E**) MEC-8 mediates both cassette exon skipping and inclusion with roughly equal frequency in both whole animals and touch neurons. (**F**) Strong inhibition of *ZK180.5* cryptic 5′ splice site by MEC-8 is observed in whole animal data, but is not discernible (as evidenced by the lack of reads on browser track) in touch neuron RNA-seq, due to the low representation of *ZK180.5* in the sequencing data. (**G**) MEC-8 regulation of *F46H5.7* cassette exon is observed in touch neuron RNA-seq, but is not discernible in whole animal RNA-seq, due to nearly-undetectable levels of exon inclusion even in wild-type conditions. (**H**) Quantification of *F46H5.7* splicing levels in all twelve RNA-seq samples. ** denotes difference between whole animal and wild-type touch neuron splicing, * denotes difference between wild-type and *mec-8(-)* touch neuron splicing (unpaired t-test, *P* < 0.05). (**I**) Seven (out of thirteen) MEC-8-regulated splicing events detected in touch neurons (see Supplementary Figure S2 for gene names) follow a pattern in which MEC-8 is responsible for a shift away from the ‘default’ splicing pattern represented in the whole animal, toward a unique touch-neuron specific isoform.

Having obtained deep-sequenced libraries of wild-type and *mec-8* mutant touch neurons, we next determined which splicing events are under the regulatory control of MEC-8 in touch neurons. In parallel we also performed whole-animal sequencing on undissociated wild-type and *mec-8* mutants to determine which splicing events are under the regulatory control of MEC-8 in all cells. Whole animal transcriptomes reveal dozens of dysregulated splicing events in *mec-8* mutants (Percent Spliced In cutoff of |ΔPSI| > 15% and *q* value < 0.05), while touch neuron transcriptomes reveal a narrower list of 13 MEC-8-regulated isoforms (Figure 2A, B, Supplemental Table). Only 5/13 (38%) of the MEC-8-regulated events in touch neurons were also identified in the whole animal analysis (Figure 2C). The types of alternative splicing regulated by MEC-8 are broadly similar in both whole animal and touch neuron transcriptomes, with cassette exons and 5′ splice sites constituting > 60% of all detected differential splicing events (Figure 2D). Among cassette exons, MEC-8 mediates both exon inclusion and exon skipping with roughly equal frequency (Figure 2E).

Most MEC-8-regulated isoforms identified from whole animal transcriptomes are not identified in touch neuron transcriptomes (Figure 2C). One reason for this is low coverage of the target transcript in touch neuron transcriptomes. For example, whole animal transcriptomes reveal that MEC-8 is necessary to repress a cryptic 5′ splice site in the *ZK180.5* gene, but this is not detected in the touch neuron dataset, which has far fewer reads covering the *ZK180.5* gene (Figure 2F). This is in accordance with experiments reporting strong expression of *ZK180.5* in hypodermis (24), highlighting that our cell-specific transcriptomic approach permits specific interrogation of MEC-8 isoform networks in touch neurons, to the exclusion of other tissues where MEC-8 is also expressed (e.g. the hypodermis).

Similarly, most MEC-8-regulated isoforms identified in touch neuron transcriptomes are not identified in whole animal transcriptomes (Figure 2C). One example of this is in the gene *F46H5.7*, which harbors an exon that is partially included in touch neurons. Inclusion of this exon requires MEC-8 (Figure 2G). In whole animal transcriptomes, however, this exon is nearly undetectable even under wild-type conditions (Figure 2G, H). A likely explanation for this observation is that the exon-included isoform is present only in touch neurons, while many other cells express the exon-skipped isoform. Indeed, a common theme among genes regulated by MEC-8 in touch neurons is that MEC-8 establishes touch-neuron specific isoforms distinct from those found in other cell types (Figure 2I). As a consequence, splicing patterns of these genes in *mec-8* mutant touch neurons more closely resemble wild-type cells of other cell types, while wild-type touch neurons exhibit unique MEC-8-mediated splicing patterns (Figure 2I).

### MEC-8 stimulates novel isoform of cytoskeletal gene *elp-1/EMAP* in touch neurons

**Figure 3.**
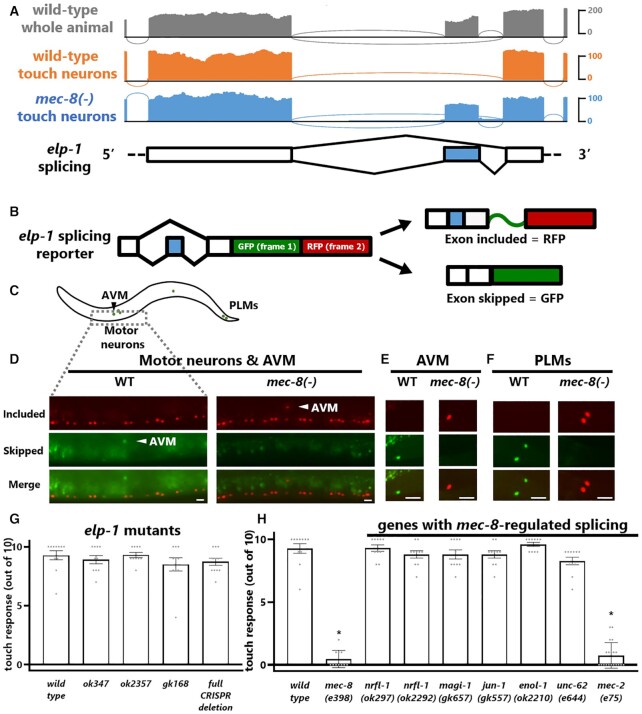
MEC-8 stimulates novel isoform of cytoskeletal gene *elp-1/EMAP* in touch neurons. (**A**) Exon #5 in the *elp-1* gene (shaded in blue below in this vignette) annotated as a constitutive exon is completely skipped in wild-type touch neurons, and this skipping requires MEC-8. (**B**) Schematic of two-color fluorescent splicing reporter. A + 1 nt frameshift is artificially introduced into the alternative exon so that inclusion vs. skipping of the exon will lead to RFP (exon inclusion, GFP is read out-of-frame with no stop codons) or GFP (exon skipped, GFP is read in frame followed by stop codon). (**C**) Worm schematic highlighting regions imaged in D-F. (**D**) *elp-1* splicing reporter driven by pan-neuronal *rgef-1* promoter. Most neurons, including the ventral nerve cord pictured here, express RFP (exon included) only in wild-type animals. Touch neurons, including the AVM pictured here, express GFP (exon skipped) only. In a *mec-8(smu1)* full deletion mutant, splicing in the ventral nerve cord is unaffected, while in the AVM splicing changes from completely skipped (GFP) to completely included (RFP). Scale bar represents 10 μm. (E, F) *elp-1* splicing reporter driven by touch-neuron specific *mec-3p* reveals similar patterns for both anterior (**E**) and posterior (**F**) touch neurons, in which wild-type touch neurons express only the skipped isoform, while *mec-8(csb22)* full deletion mutant touch neurons express only the included isoform. Scale bar represents 10 μm. (**G**) *elp-1* mutants show no discernible touch sensation behavioral defects (all *P*-values > 0.05, one-way ANOVA compared to wild type). (**H**) Loss-of-function alleles for most MEC-8-regulated genes do not result in touch sensation behavioral defects, with the strong exception of *mec-2(e75)*. Asterisk indicates *P* < 0.05, one-way ANOVA compared to wild type.

A striking example of MEC-8 establishing unique splicing patterns in touch neurons can be found in *elp-1*, the *C. elegans* homologue of the microtubule-associated gene EMAP. Our touch neuron transcriptomes reveal alternative splicing of an exon currently annotated as a constitutive exon (Figure 3A). In wild-type whole animals the exon is partially included and partially skipped. In wild-type touch neurons the exon is completely skipped. This exon skipping depends on MEC-8, as *mec-8* mutant touch neurons exhibit a dramatic switch from exon skipping to exon inclusion (Figure 3A, Supplementary Figure S3A). Therefore, our sequencing results reveal that MEC-8 is required to establish a novel exon-skipped isoform of *elp-1* in touch neurons.

To further examine the regulation of *elp-1* isoforms we generated two-color splicing reporters, which enable *in vivo* visualization of splicing in *C. elegans* with single-cell resolution (25–28). Engineering a + 1 nucleotide translational frameshift into the alternative exon of a transgenic minigene results in a two-color readout of exon inclusion (RFP) versus exon skipping (GFP) (Figure 3B). An *elp-1* splicing reporter driven by a pan-neuronal promoter reveals exon inclusion in most neurons, for example the motor neurons of the ventral nerve cord (Figure 3C, D), while touch neurons express only the skipped isoform (Figure 3D, Supplementary Figure S3B). In *mec-8* mutants, the splicing pattern in the ventral nerve cord is unchanged, but touch neurons undergo a dramatic shift from expressing only the skipped isoform to expressing only the included isoform (Figure 3D, Supplementary Figure S3B). Similar results are obtained with an *elp-1* splicing reporter driven by a touch-neuron promoter (Figure 3E-F). Wild-type touch neurons express only the skipped isoform, while *mec-8* mutant touch neurons express only the included isoform (Figure 3E-F). These findings are in agreement with our transcriptomic data, indicating that MEC-8 establishes a unique non-canonical isoform of *elp-1* specifically in touch neurons.

We next tested whether *elp-1* isoforms might play a functional role in touch neuron function. Touch neurons are rich in microtubules, and mutant alleles in the microtubule-binding *elp-1/*EMAP gene were reported to result in modest touch-sensation defects (29). However, using existing loss-of-function alleles, we do not detect any appreciable loss of touch sensation in *elp-1* mutants (Figure 3G, Supplementary Figure S3C). We also generated a null CRISPR/Cas9-mediated deletion of the entire *elp-1* coding sequence, which also does not affect touch sensation (Figure 3G). We therefore conclude that either *elp-1* does not play a major role in touch sensation, or its role is functionally redundant with additional touch-neuron genes.

We took a similar approach to test for functional roles of other MEC-8-regulated genes in touch sensation. Among the thirteen touch neuron MEC-8-regulated genes, mutant alleles exist for seven (including *elp-1*). We tested these mutants and found that most have no discernable effect on touch sensation (Figure 3H). A hypomorphic allele of the transcription factor *unc-62* causes mild, but not statistically-significant, defects in touch sensation (Figure 3H). Previous studies indicate that null *unc-62* mutants are embryonic lethal, and exhibit gene expression defects in a subset of embryonic touch neurons (30). This suggests that *unc-62* might play a role in touch neuron function. In contrast with the mild defects presented above, we observe a clear touch-sensation phenotype in a mutant of one MEC-8-regulated gene, *mec-2*/Stomatin (Figure 3H), which we further investigate below.

### Non-canonical *mec-2/*Stomatin isoform is expressed in non-mechanosensory neurons

**Figure 4.**
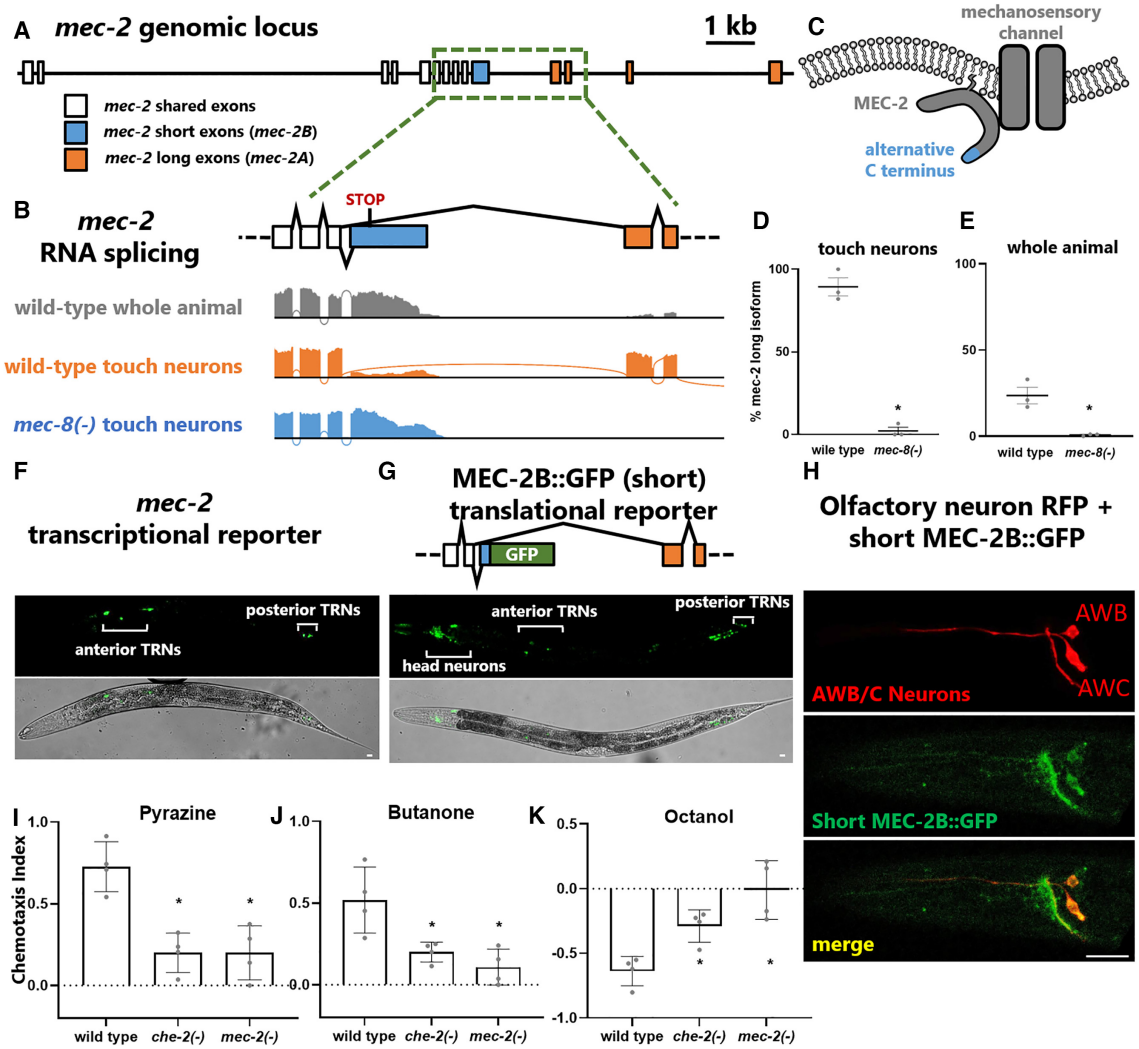
Non-canonical *mec-2/Stomatin* is present in non-mechanosensory neurons, and *mec-2* is required for non-mechanosensory behaviors. (**A**) Entire genomic locus of *mec-2*, with non-canonical short isoform depicted in blue and canonical long isoform in orange. (**B**) wild-type touch neurons express primarily the canonical *mec-2A* (‘long’) isoform, while both *mec-8* mutant touch neurons and, surprisingly, wild-type whole animals, express primarily the *mec-2B* (‘short’) isoform, encoding both a stop codon and predicted 3′ cleavage and polyadenylation sequence, see also Supplementary Figure S4B. (**C**) The alternatively-spliced C terminus is depicted in context of its putative location at the membrane and association with the mechanosensory channel. (D, E) Quantification of *mec-2* isoform abundance (asterisk indicates *P* < 0.05, unpaired *t*-test) in touch neurons (**D**) and whole animals (**E**) in both wild-type and *mec-8* mutant conditions across all twelve RNA-seq samples. (**F**) *mec-2p::GFP* (using 2.5 kb upstream of the *mec-2* start codon) expression is detectable only in touch neurons. (**G**) MEC-2B::GFP expression from a full-length translational fosmid fusion (including an additional 7.5 kb of potential upstream regulatory sequence and 8 kb of potential downstream regulatory sequence) is detectable in many neurons in addition to touch neurons. Scale bar represents 10 μm. (**H**) Translational MEC-2B::GFP fusion is expressed in olfactory AWB and AWC neurons. (**I**-**K**) *mec-2(e75)* mutants are defective in olfaction to a variety of olfactory cues, including 1 mM pyrazine, 1:1000 butanone and 1:1000 octanol (*n* = 4 independent trials, asterisk indicates *P* < 0.05, one-way ANOVA compared to wild type).

In addition to having a severe touch-sensation phenotype (Figure 3H), *mec-2* is also the strongest MEC-8-regulated splicing event in our dataset (ΔPSI = 87%). *mec-2/*Stomatin is a conserved component of the mechanosensory transduction machinery, and is essential for proper function of mechanosensory channels in both worms and mice (31,32). In *C. elegans*, the *mec-2* gene contains an alternative 3′ splice site resulting in the production of an mRNA encoding either a long (*mec-2A*) or short (*mec-2B*) C terminus (Figure 4A–C, Supplementary Figure S4B). Our data show that MEC-8 strongly regulates this splicing choice, with wild-type touch neurons expressing mostly the long isoform, and *mec-8* mutant touch neurons expressing mostly the short isoform (Figure 4B–D). This is consistent with previous genetic studies defining the long *mec-2* isoform as the functional ‘canonical’ isoform, and requiring MEC-8 for its expression (33).

Surprisingly, however, analysis of wild-type whole animal RNA-seq indicates that the majority of *mec-2* is present in the form of the non-canonical short *mec-2B* isoform (Figure 4B, E). Previously it was assumed that since *mec-2* is expressed only in touch neurons, and since the long *mec-2A* isoform predominates in touch neurons, therefore the short *mec-2B* isoform likely represents an aberrant non-functional isoform produced in the absence of MEC-8 (33). In contrast, our data reveal substantial expression of *mec-2* in non-touch neuron cells, and indicate that the vast majority of *mec-2* is present in the non-canonical short isoform.

To further examine whether the non-canonical short *mec-2* isoform is expressed in additional cell types, we used *in vivo* transgenic reporters. We generated a transcriptional *mec-2* reporter in which GFP is driven by a 2.5 kb *mec-2* promoter. We also generated a translational fosmid-based reporter containing the entire *mec-2* locus, including introns and thousands of nucleotides of potential upstream and downstream regulatory elements, with the short *mec-2B* isoform fused to GFP (Figure 4F, G). The transcriptional reporter recapitulated the canonical expression pattern of *mec-2*, with expression restricted to touch neurons (Figure 4F). On the other hand, the *mec-2B* (short) translational reporter revealed expression in touch neurons as well as many additional neurons in the head and tail (Figure 4G). This corroborates our RNA-seq observations that the non-canonical short *mec-2B* isoform is the predominant form of *mec-2* expressed in wild-type animals, with expression in both touch neurons and additional neuron types.

### *mec-2* is expressed in olfactory neurons and is required for olfaction

Given the large number of head neurons expressing the short isoform MEC-2B::GFP, it was difficult to identify expression in specific neuronal classes. However, we did observe expression in neuronal processes emanating from the anterior region of the pharyngeal bulb and extending to the nose tip, suggesting that they might belong to sensory neurons of the amphid (34). To test whether the short *mec-2B* isoform is expressed in specific amphid neurons, we labeled the amphid AWB and AWC olfactory neurons with an *odr-1p*::RFP reporter. MEC-2B::GFP expression is clearly visible in both AWB and AWC neuronal cell bodies, as well as their axons and dendrites (Figure 4H). Together these data indicate that the short *mec-2B* isoform is present in at least two different types of sensory neurons: mechanosensory touch neurons and chemosensory olfactory neurons.

Since we detected short *mec-2B* isoform expression in olfactory neurons, we next asked whether *mec-2* plays a functional role in olfactory behavior. We found that *mec-2* loss-of-function mutants are not only defective in mechanosensation, but also in olfaction (Figure 4I–K, Supplementary Figure S4A). We performed chemotaxis assays on populations of worms to test their ability to respond to both attractive and repulsive odors. *mec-2* mutants are strongly chemotaxis defective to both attractive and repulsive odors, with deficits equal to those of control chemosensation-deficient *che-2* mutants (Figure 4I–K). Together these results reveal surprising expression of a short *mec-2* isoform in many neurons, including both touch neurons and olfactory neurons, and demonstrate a functional requirement for *mec-2* in both touch sensation and olfaction.

### Endogenous *mec-2* isoforms are differentially expressed in different neuron types

**Figure 5.**
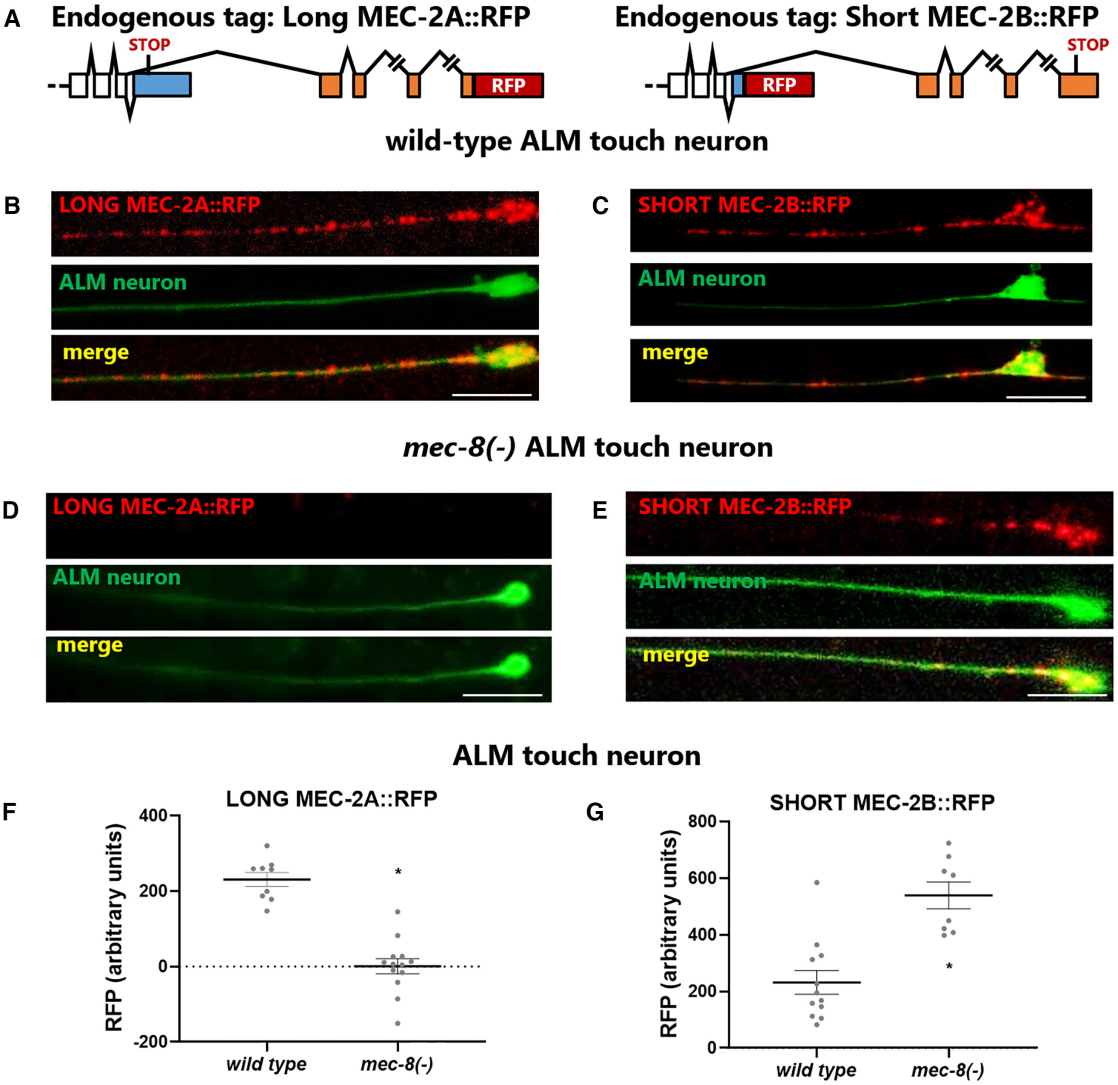
Endogenous *mec-2* isoforms are differentially expressed and differentially affected by *mec-8* mutation. (**A**) Two complementary endogenous strains were created using CRISPR/Cas9 genome editing: an RFP-tagged long *mec-2A* strain and an RFP-tagged short *mec-2B* strain. (**B**) MEC-2A::RFP is expressed in ALM touch neuron in a punctate pattern throughout both cell body and neurite. (**C**) MEC-2B::RFP is also expressed in ALM in punctate pattern throughout cell body and neurite. (**D**) *mec-8* mutation results in loss of MEC-2A::RFP expression. (**E**) *mec-8* mutation does not result in loss of MEC-2B::RFP expression. (F, G) Quantification of RFP intensity (asterisk indicates *P* < 0.05, unpaired t-test) demonstrating that *mec-8* mutation causes (**F**) a decrease in MEC-2A::RFP expression and (**G**) a concomitant increase in MEC-2B::RFP expression in ALM touch neurons. Scale bars represent 5 μm.

To determine the endogenous neuron-specific expression patterns of both *mec-2* isoforms, we used CRISPR/Cas9 to endogenously tag each isoform with a C-terminal translational fusion (Figure 5A). Tagging the canonical long *mec-2A* isoform with RFP revealed strong expression in the touch neurons, with puncta visible in both the cell body and throughout the neurite (Figure 5B, Supplementary Figure S5), in accordance with previous imaging using antibody staining (35). Endogenously-tagged short *mec-2B* isoform is also detectable in touch neurons, with a similar punctate expression (Figure 5C, Supplementary Figure S5A).

In agreement with our transgenic MEC-2B::GFP overexpression results above (Figure 4G), we find endogenous short isoform MEC-2B::RFP expressed abundantly in many head and tail neurons in addition to touch neurons (Supplementary Figure S5B). This is not the case with endogenously-tagged long isoform MEC-2A::RFP, where expression is limited to mechanosensory neurons (Supplementary Figure S5B).

We next crossed the endogenous isoform-specific tagged strains with a *mec-8* mutant. The long isoform MEC-2A::RFP is reduced to undetectable levels in a *mec-8* mutant, while the short isoform MEC-2B::RFP exhibits increased fluorescence (Figure 5D–G, Supplementary Figure S5F, G). These results demonstrate that endogenous long *mec-2A* isoform expression is specific to mechanosensory neurons, and is dependent on MEC-8 for its expression, while the short *mec-2B* isoform is expressed in both touch neurons as well as many other neuron types, and its expression does not require MEC-8.

### *mec-2* isoform-specific function in mechanosensation and chemosensation

**Figure 6.**
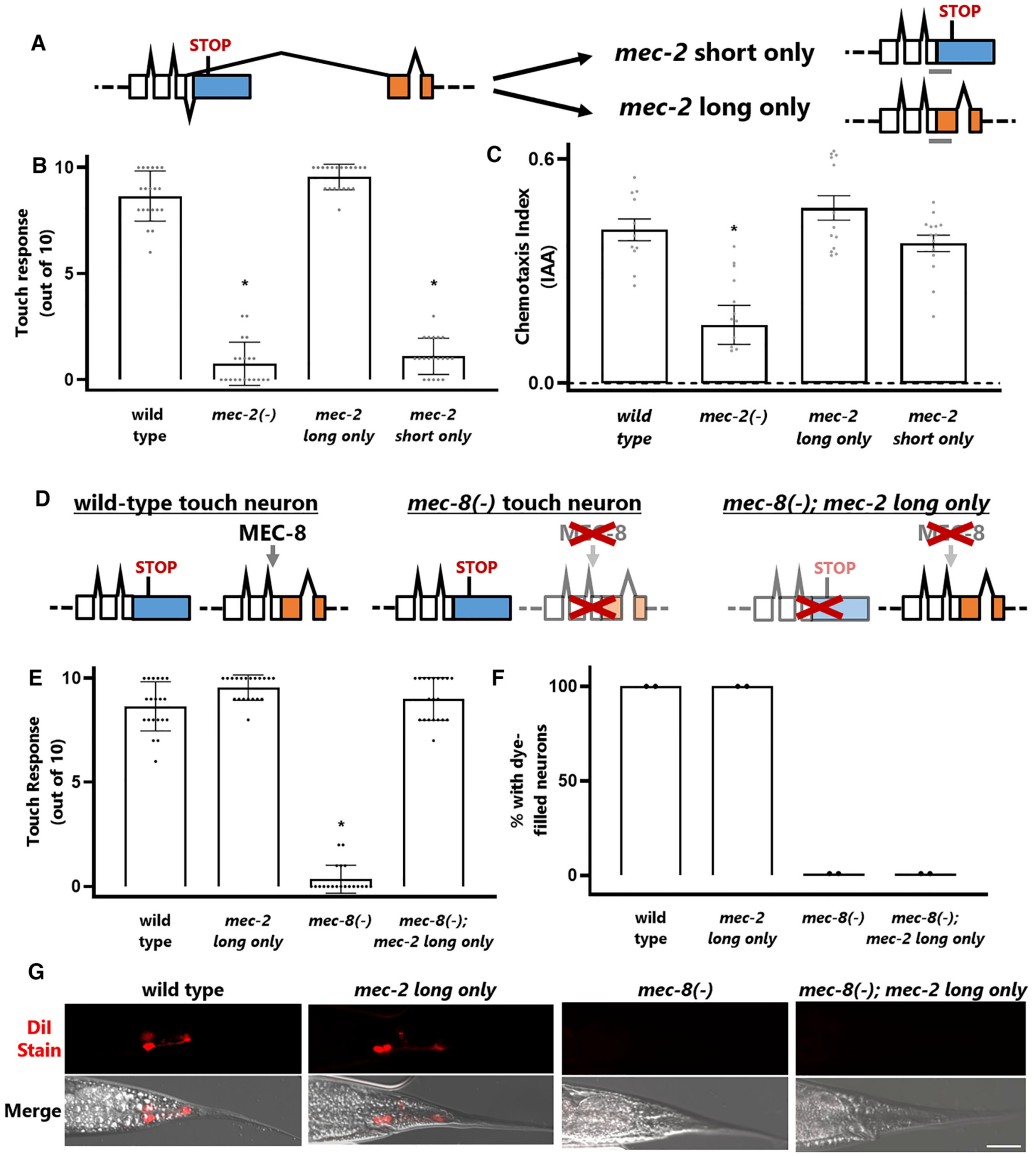
*mec-2 i*soform-specific function and rescue of *mec-8(-)* touch sensation defects. (**A**) Schematic of CRISPR/Cas9 genome edits forcing expression of either the long or short *mec-2* isoforms by fusing the alternative exon directly to the final common upstream exon and deleting the intervening sequence. (**B**) Forced expression of the long *mec-2* isoform results in normal touch sensation, but forced expression of the short isoform results in touch-sensing defects indistinguishable from a *mec-2* loss-of-function mutant. (**C**) Forced expression of either the long or short isoform of *mec-2* results in wild-type levels of olfaction, IAA 1:100,000 dilution. (**D**) Schematic representation of the double mutant strain in which simultaneously *mec-8* is mutated but the *mec-2* long isoform is genetically restored. (**E**) Forced expression of the long *mec-2* isoform completely restores *mec-8* mutant touch defects. (**F**) Forced expression of the long *mec-2* isoform has no effect on *mec-8* mutant phasmid neuron dye-filling defects. (**G**) Representative images of phasmid dye-filling defects. Scale bar represents 10 μm. Asterisks indicate *P* < 0.05, one-way ANOVA, compared to wild type.

Having established that *mec-2* isoforms are differentially expressed in different types of sensory neurons, we next asked whether they are functionally distinct. Specifically, we tested whether forced expression of one isoform can substitute for loss of the other isoform with respect to sensory behaviors. We generated endogenous isoform-specific *mec-2* mutants using CRISPR/Cas9 to force expression of either the short isoform only or the long isoform only (Figure 6A).

We then tested the isoform-specific mutants for mechanosensation and chemosensation. Touch assays reveal clear isoform-specific function of *mec-2* (Figure 6B). Animals with forced expression of the canonical long *mec-2A* isoform sense touch as well as wild type, while animals with forced expression of the non-canonical short *mec-2B* isoform are touch-insensitive and indistinguishable from *mec-2* loss-of-function mutants (Figure 6B). This indicates that the long *mec-2* isoform is the relevant isoform for mechanosensation, and that the short *mec-2* isoform cannot functionally substitute.

In contrast, chemotaxis assays reveal that animals with forced expression of either *mec-2* isoform respond to olfactory cues at wild type levels (Figure 6C). This indicates that although the long *mec-2* isoform is not expressed at detectable levels in olfactory neurons, it can functionally substitute for the short *mec-2* isoform when ectopically expressed. Together these results reveal differential expression and function for *mec-2* isoforms in different sensory neurons. The long isoform is present in mechanosensory neurons and is required for mechanosensation. The short isoform is present in olfactory neurons, but either isoform is capable of functioning in olfaction when artificially expressed.

### Restoring *mec-2* long isoform rescues mechanosensation in *mec-8* mutants

Given that the long *mec-2A* isoform is essential for mechanosensation, and that the *mec-8* RNA binding protein is required for *mec-2A* long isoform expression in touch neurons, we wondered whether aberrant *mec-2* splicing could partially explain the mechanosensory defects in *mec-8* mutants. Our endogenous isoform-specific mutants allowed us to test this question, one which is usually difficult or impossible to test: among the many targets in a splicing network controlled by an RNA binding protein, which are functionally relevant?

We crossed our endogenous *mec-2* long only strain with a *mec-8* loss-of-function mutant, thereby restoring expression of a single *mec-8* target isoform (*mec-2* long), while leaving all other *mec-8* targets dysregulated (Figure 6D). Remarkably, restoring *mec-2* long isoform expression completely rescues the mechanosensory defects in a *mec-8* mutant, as the *mec-8; mec-2 long only* double mutant is indistinguishable from wild type (Figure 6E). This indicates that although there are dozens of isoforms affected by *mec-8* loss of function, the primary functionally-relevant *mec-8* target in touch neurons is *mec-2*.

On the other hand, *mec-8* is also essential for the function of other neurons and cell types. For example, *mec-8* mutants exhibit structural defects in the phasmid neurons (PHA and PHB) of the tail, as evidenced by the failure of phasmid neurons to uptake lipophilic dyes such as DiI (Figure 6F, G). Restoring *mec-2* long isoform expression does not rescue the *mec-8* mutant phasmid neuron defects (Figure 6F, G). These results indicate that the critical functionally-relevant MEC-8 targets differ in different neuron types. In touch neurons, *mec-2* is the primary functionally-relevant target, while phasmid neuron function depends on other *mec-8* targets besides *mec-2*.

### Two distinct long *mec-2* isoforms are expressed in touch neurons

**Figure 7.**
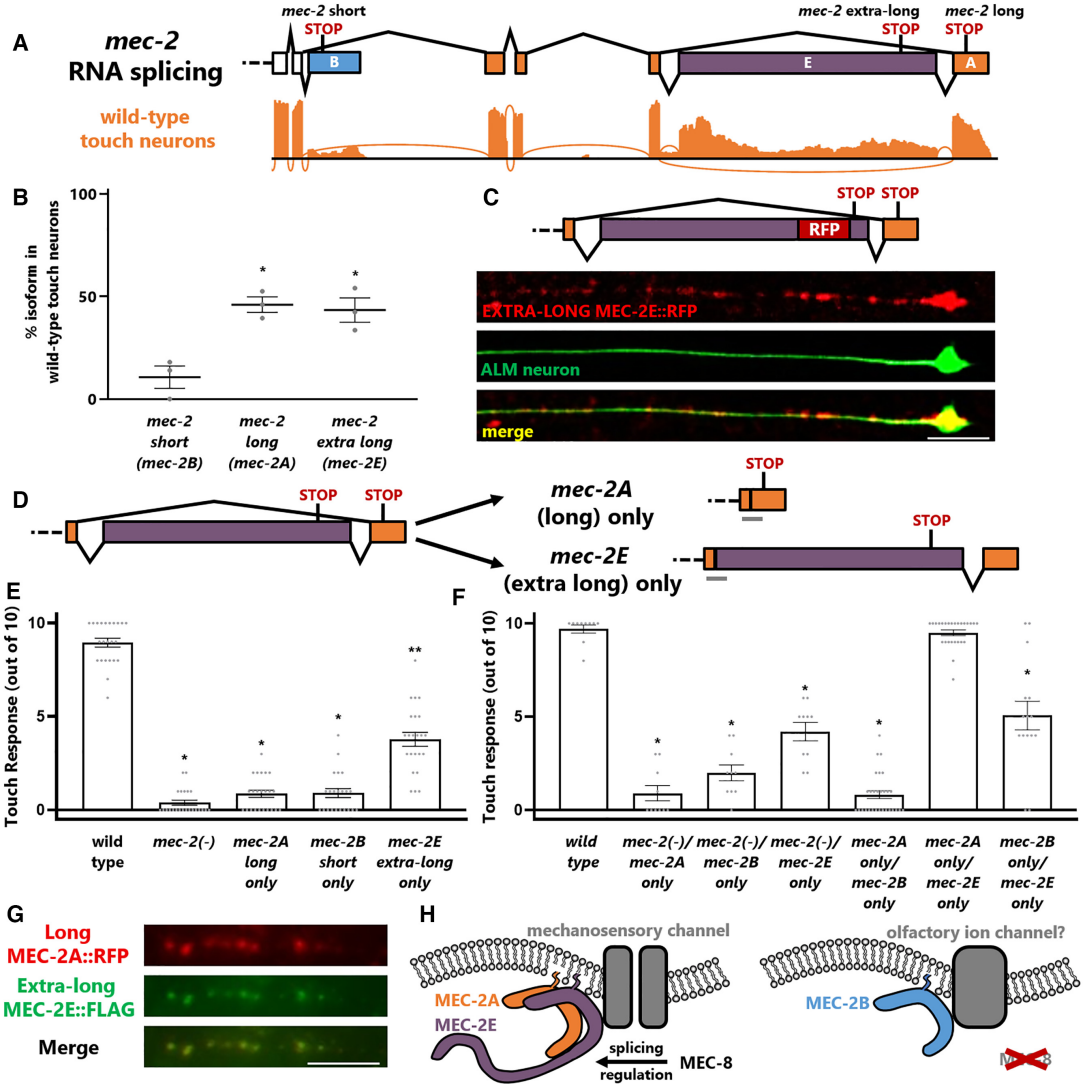
Two distinct long *mec-2* isoforms both required for touch sensation. (**A**) Wild-type touch neuron RNA-seq reveals substantial inclusion of a very large alternative cassette exon within the context of the long *mec-2* isoform. (**B**) *mec-2E* (extra long isoform) is detected at similar levels to the canonical *mec-2A* (long) isoform in our wild-type touch neuron RNA-seq. One-way ANOVA, asterisk indicates *P* < 0.05 compared to wild type. (**C**) Endogenously-tagged MEC-2E::RFP is expressed in touch neurons, and exhibits a punctate expression pattern similar to that seen for MEC-2A::RFP (see Figure 5B). Scale bar represents 5 μm. (**D**) Schematic of doubly-edited strains in which first the long isoforms are forced (see Figure 6A), then either the long (*mec-2A*) or extra long (*mec-2E*) are further force-expressed via CRISPR/Cas9 genome editing. (**E**) None of the individual *mec-2* isoforms is sufficient for touch sensation, although the extra-long *mec-2E* isoform confers a modest degree of touch sensation. One-way ANOVA, * *P* < 0.05 compared to wild type, ** *P* < 0.05 to both wild-type and *mec-2(-)*. (**F**) Compound heterozygotes reveal that only heterozygotes containing one copy of *mec-2E only* and one copy of *mec-2A only* are restored to wild-type levels of touch sensation. One-way ANOVA, asterisk indicates *P* < 0.05 compared to wild type (**G**) Endogenously-tagged long MEC-2A::RFP and endogenously-tagged (also via CRISPR/Cas9) extra-long MEC-2E::FLAG exhibit extensive punctate co-localization in individual neurites of touch neurons, as assessed by immunofluorescence. Pearson's coefficient *r* = 0.653. (**H**) Model showing that MEC-2A and MEC-2E, whose expression is facilitated by the MEC-8 RNA binding protein, together mediate touch sensation in the touch neurons, while MEC-2B alone is present in other neurons, such as the olfactory neurons.

Further inspection of wild-type touch neuron RNA-seq reveals that within the context of the long *mec-2* isoform, additional isoform diversity exists. Encoded within the final intron of the canonical *mec-2A* long isoform is an uncharacterized alternative cassette exon of unusually large size (2,340 bp). When included, this exon results in an extra-long *mec-2E* isoform roughly triple the size of the canonical long *mec-2A* isoform (Figure 7A). Despite its unusually large size (it is the second-largest alternative cassette exon we detect in our transcriptome data), and despite the mechanistic difficulty posed to the spliceosome by exons of extreme size (36), our RNA-seq data indicate that the extra-large exon is included at appreciable levels in wild-type touch neurons (Figure 7B).

To test whether the extra-long *mec-2E* isoform is translated into a stable product *in vivo*, we generated an endogenous RFP-tagged *mec-2E* strain, and found that extra-long MEC-2E::RFP is expressed specifically in touch neurons. Similar to the long MEC-2A isoform, the extra-long MEC-2E isoform is expressed in a punctate pattern in both the cell bodies and the neurites of the touch neurons (Figure 7C), and this expression is abolished in *mec-8* mutants (Supplementary Figure S6A). Unlike the short MEC-2B isoform, there is no detectable expression of extra-long MEC-2E in head neurons.

### Both long *mec-2* isoforms are required for touch sensation

To test whether the extra-long *mec-2E* isoform is functionally important, we further modified our *mec-2* long only endogenous mutants. We previously generated a mutant forcing expression of the long *mec-2* isoforms to the exclusion of the short *mec-2B* isoform (Figure 6A). We further modified this strain to force either inclusion or skipping of the large alternative cassette exon, thus forcing expression of either the long (*mec-2A*) or extra-long (*mec-2E*) isoform (Figure 7D). Either isoform is sufficient for wild-type levels of olfactory behavior (Supplementary Figure S6B). Surprisingly, however, neither isoform is sufficient for functional touch sensation (Figure 7E). Forcing expression of the canonical long *mec-2A* isoform results in animals with no touch sensitivity, indistinguishable from *mec-2* null mutants (Figure 7E). On the other hand, forcing expression of the non-canonical extra-long *mec-2E* isoform results in animals with partial touch sensitivity (Figure 7E). Together these results indicate that while forcing the long and extra-long isoforms *mec-2A/E* at the expense of the short *mec-2B* isoform results in wild-type levels of mechanosensation (Figure 6B), neither of the longer isoforms alone is sufficient for mechanosensation (Figure 7E).

Since the long *mec-2A* and extra-long *mec-2E* isoforms are both expressed in touch neurons in roughly equal proportions (Figure 7B), and since neither isoform is sufficient for wild-type levels of mechanosensation (Figure 7E), we next asked whether simultaneously forcing expression of both *mec-2A* and *mec-2E* is sufficient for touch sensation. To do this we created compound heterozygotes containing one genomic copy of each isoform-specific allele (*mec-2A/mec-2E*). Remarkably, these compound heterozygotes are fully touch sensitive, indistinguishable from wild-type animals (Figure 7F). In contrast, compound heterozygotes including either a null allele or the short *mec-2* isoform (*mec-2B*) remain touch-insensitive (Figure 7F). These results indicate that both the long *mec-2A* and extra-long *mec-2E* isoforms are simultaneously required for touch sensation.

The long *mec-2A* and extra-long *mec-2E* forced-expression alleles therefore exhibit ‘allelic complementation’ or ‘intragenic complementation.’ This type of genetic result often indicates that the protein products of the gene in question form a multimeric complex *in vivo* (37). If this is the case with *mec-2*, our results suggest that functional heteromultimers are formed by long MEC-2A and extra-long MEC-2E. In contrast, neither homomultimers nor heteromultimers formed with the short MEC-2B isoform are functional. As an additional test of this hypothesis, we endogenously tagged both long MEC-2A and extra-long MEC-2E isoforms in the same strain, and observed extensive punctate co-localization in neurites (Figure 7G, Supplementary Figure S6C–E). This is consistent with the hypothesis that MEC-2A and MEC-2E isoforms function as heteromers.

Finally, given the diversity of *mec-2* isoform functions, we asked whether the three *mec-2* isoforms encode unique functional protein domains that could explain their unique functions. Each of the three isoforms encodes the stomatin domain and membrane-interaction domain characteristic of the Stomatin family of proteins (Supplementary Figure S7A). The alternative C-terminal isoforms, however, harbor no predicted protein domains (Supplementary Figure S7A). Protein disorder prediction by PrPROT suggests that the C terminus of *mec-2* is intrinsically disordered. The short *mec-2B* and long *mec-2A* encode a short stretch of predicted disorder, while the extra-long *mec-2E* isoform encodes a long stretch of predicted disorder (Supplementary Figure S7A). Intrinsically-disordered termini of other membrane-associated proteins have been implicated in protein-protein interactions and functional modulation (38). We therefore speculate that *mec-2* isoforms have unique roles in protein-protein interaction and recruitment, resulting in critical regulation of the function of both mechanosensory and chemosensory neurons (Figure 7H).

## DISCUSSION

Using a combination of cell-specific transcriptomics, precision genome editing, and behavioral analysis, we identify regulatory and functional activities of the RNA binding protein MEC-8 in a specific neuron type, the *C. elegans* touch neurons. Our approach of obtaining deep sequencing libraries from thousands of sorted touch neurons has unique advantages over standard single-cell sequencing: increased transcript coverage and uniformity enables splicing analysis throughout the gene body and sensitivity to detect multiple isoforms of a gene in a single cell type. This is exemplified by our discovery of two *mec-2* isoforms co-expressed in touch neurons, both of which are required for mechanosensation. Our approach also has unique advantages over whole-animal or whole-tissue sequencing, allowing us to focus on the regulatory role of MEC-8 in touch neurons, to the exclusion of its role in other neurons and tissues. This is exemplified by the identification of a small handful of MEC-8-regulated targets in touch neurons, including a single target (*mec-*2) that fully accounts for the *mec-8* mutant mechanosensory phenotype. Historically, identifying functionally-relevant target(s) among the dozens to hundreds regulated by a given RNA binding protein or transcription factor usually proves difficult or impossible (39,40). As such, our approach provides a potential template for future studies aimed at elucidating functionally-relevant regulatory targets in specific cell types.

### Non-canonical *mec-2B* isoform functions in chemosensation but not mechanosensation

We find that the short *mec-2B* isoform is the predominant species of *mec-2* in non-touch-neuron cells. Its expression is confined mostly to neurons of the head and tail ganglia. While many of these neurons remain unidentified, we find strong *mec-2B* expression in specific olfactory neurons in the head. We also find that *mec-2* is required for olfactory behavior. However, while *mec-2B* is unable to confer touch sensation when artificially expressed in touch neurons, *mec-2A* and *mec-2E* isoforms are able to confer olfaction when ectopically expressed in olfactory neurons. Therefore, while only *mec-2B* is normally expressed in olfactory neurons, all three isoforms are functional when ectopically expressed.

What then is the utility of the short *mec-2B* isoform? One possibility is that *mec-2B* is required for functions not yet uncovered by our standard laboratory experiments. Another possibility is that the short *mec-2B* isoform represents an energetically favorable version of *mec-2*. The energetic cost of unnecessary RNA synthesis, processing, and translation can reach levels sufficient to confer selective disadvantages (41). Since *mec-2B* functions as well as the longer *mec-2A* or *E* isoforms in olfactory neurons, selecting the *mec-2B* isoform avoids thousands of unnecessary nucleotides of transcription, RNA processing, and translation. However, since the *mec-2B* isoform is non-functional with regard to mechanosensation, the longer *mec-2* isoforms must be synthesized in touch neurons, which is mediated by the RNA binding protein MEC-8.

The role of MEC-8 in directing cell-specific expression of long *mec-2* isoforms appears to rely on partially-overlapping expression patterns. Many cell types express *mec-8* alone or *mec-2* alone. Only cells expressing both genes (*i.e*. the touch neurons) generate the long *mec-2* isoforms. Other neurons expressing *mec-2* but not *mec-8* produce the short *mec-2B* isoform. Additionally, other RNA binding proteins could in principle provide further levels of co-regulation to *mec-2* splicing. For example, the splicing factors *smu-1, smu-2, sym-2*, and *exc-7* have all been demonstrated to genetically interact with *mec-8* (42–44). Existing data do not reveal any clear role for these factors in *mec-2* splicing or in touch neuron-specific splicing (22,45), but systematic analyses have not directly addressed this question.

### Multiple *mec-2* isoforms function together in touch sensation

Our RNA-seq data indicate that the canonical *mec-2* long isoform, which is stimulated by MEC-8, is further diversified in touch neurons by alternative inclusion or skipping of a very large exon. This exon is interesting from a regulatory perspective, as extremely large exons tend to be spliced with lower efficiency (36). It is also interesting from a functional perspective, since the included isoform coding sequence is three times larger than the skipped isoform, which could conceivably alter its localization, binding partners, or other functional aspects. Nevertheless, it is endogenously expressed in a punctate pattern in touch neurons, just as the canonical long *mec-2A* isoform. In fact, it is the only isoform that confers partial touch sensitivity on its own when genetically force-expressed, indicating that it is a *bona fide* member of the touch-sensing molecular pathway. Moreover, the *mec-2E* sequence is conserved among distant nematode species (Supplementary Figure S7B), suggesting functional importance over evolutionary time scales.

*mec-2* and its homologues have previously been suspected to form multimers, based on *in vitro* biochemical experiments in mammalian cells (46), electron microscopy of *C. elegans* touch neurons (35), and the observation that certain loss-of-function *mec-2* alleles exhibit intragenic complementation (10). Likewise, in *Xenopus* oocytes, MEC-2 is able to self-associate, and requires its C-terminus to do so (47). We now extend these findings and propose that MEC-2A and MEC-2E isoforms function together as heteromers. A potentially related phenomenon has been noted in mammalian cells, where two distinct *mec-2* gene homologues with divergent C termini (stomatin and STOML3) were found to colocalize in cultured cells (48).

Our proposal that the two long *mec-2* isoforms function together as heteromers in a single neuron type is conceptually different from cases of isoform-specific function in specific neurons where different isoforms with different functions are expressed in different neuron types (49,50). In most cases of cell-specific alternative splicing, isoform-specific functions remain undetermined (40,51). In cases where distinct functions have been elucidated, the alternative isoforms often have distinct functions in distinct cell types (52). For example, in the classic case of calcitonin/CGRP, alternative splicing leads to the production of two peptides with strikingly different functions in different cell types: calcitonin in thyroid C cells versus the CGRP neuropeptide in neurons (53).

In contrast, we find that within a single neuron type, different *mec-2* isoforms collaborate to produce a functional output. It will be interesting to determine whether such collaboration between isoforms constitutes a common theme as single-cell transcriptomic regulation and function are probed with increasing resolution. Current single-cell sequencing technologies are limited in their ability to simultaneously detect multiple isoforms of a single gene, which can lead to the erroneous conclusion of binary splicing choices in which only one isoform is expressed per cell (7). This highlights a strength of cell-specific deep transcriptomes, which are capable of detecting multiple isoforms of a single gene with cell-specific resolution. In this study deep touch neuron transcriptomes, coupled with powerful genetics and behavioral assays available in *C. elegans*, reveal both regulation and function of alternative splicing in specific neurons.

## DATA AVAILABILITY

The sequencing data generated in this study are available at the NCBI SRA archive (PRJNA752629) and GEO (GSE184149).

## Supplementary Material

gkab1134_Supplemental_Files
