# Supplementary material for: Sensory neuron transcriptomes reveal complex neuron-specific function and regulation of mec-2/Stomatin splicing
Source: Nucleic Acids Res. 2021 Dec 8;50(5):2401–16. doi: 10.1093/nar/gkab1134 (PMC8934639; doi:10.1093/nar/gkab1134)
Supplement: gkab1134_Supplemental_Files [file gkab1134_supplemental_files.zip › SUPPLEMEMTAL FIGURES.pdf]

SUPPLEMEMTAL FIGURES

A

	#neurons sorted	Uniquely mapped reads number	Uniquely mapped reads %
wt_TRN1	17,000	27,133,431	35.94%
wt_TRN2	18,387	28,949,167	51.49%
wt_TRN3	20,954	15,926,736	32.12%
GFP-_sorted1	-	28,949,167	51.49%
GFP-_sorted2	-	31,367,741	44.85%
GFP-_sorted3	-	23,408,107	28.98%
mec8_TRN1	2,000	34,535,015	63.59%
mec8_TRN2	14,711	31,367,741	44.85%
mec8_TRN3	5,138	23,408,107	28.98%
wholeworm_wt1	-	36,968,769	56.35%
wholeworm_wt2	-	33,931,980	53.42%
wholeworm_wt3	-	39,061,623	59.67%
wholeworm_mec8_1	-	40,431,156	60.19%
wholeworm_mec8_2	-	37,757,433	58.95%
wholeworm_mec8_3	-	46,977,080	59.51%

B

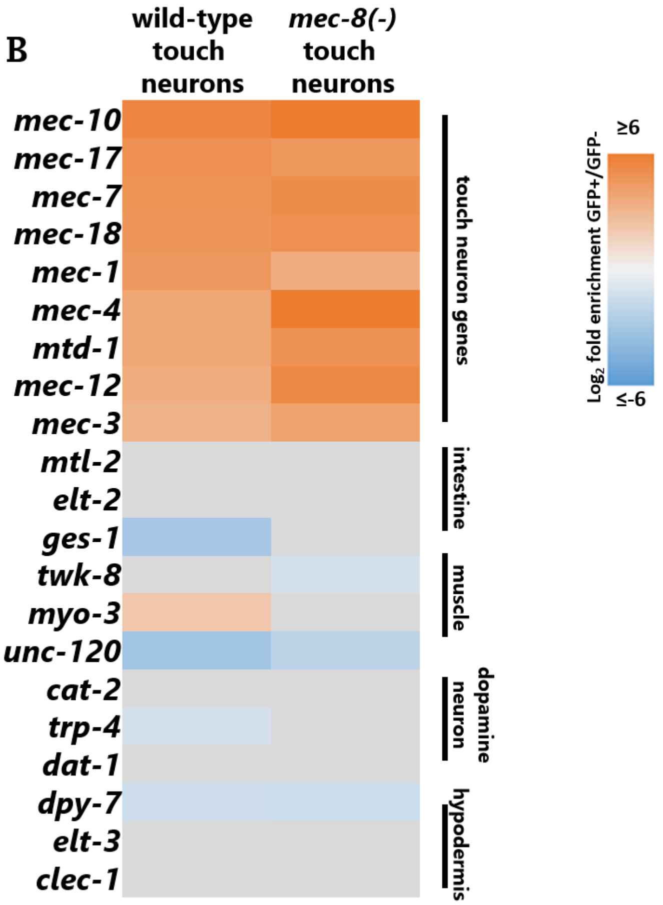

Figure S1: Sorting and sequencing of wild-type and *mec-8(-)* touch neurons. (A) Number of cells sorted and number of uniquely-mapped reads in sorted neuron and whole worm experiments. (B) Heatmap of tissue-specific and cell-specific gene expression enrichment in sorted neuron populations. As in Fig 1F but with additional detail on individual genes.

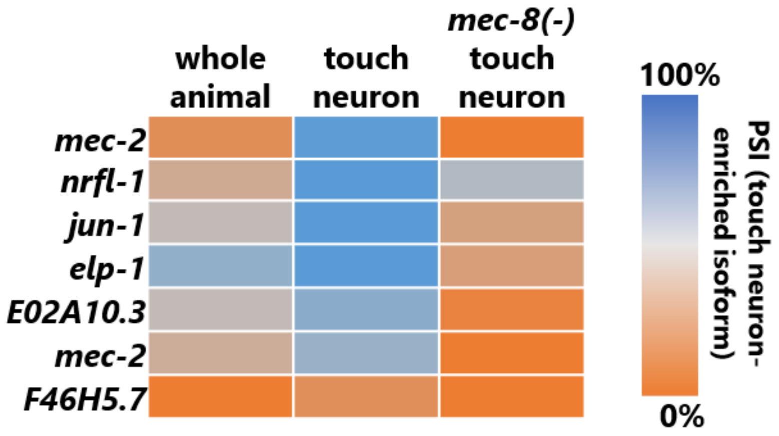

Figure S2: *mec-8*-mediated unique isoforms in touch neurons. Underlying gene names provided for heatmap of *mec-8*-mediated unique isoforms in touch neurons displayed in Figure 2I.

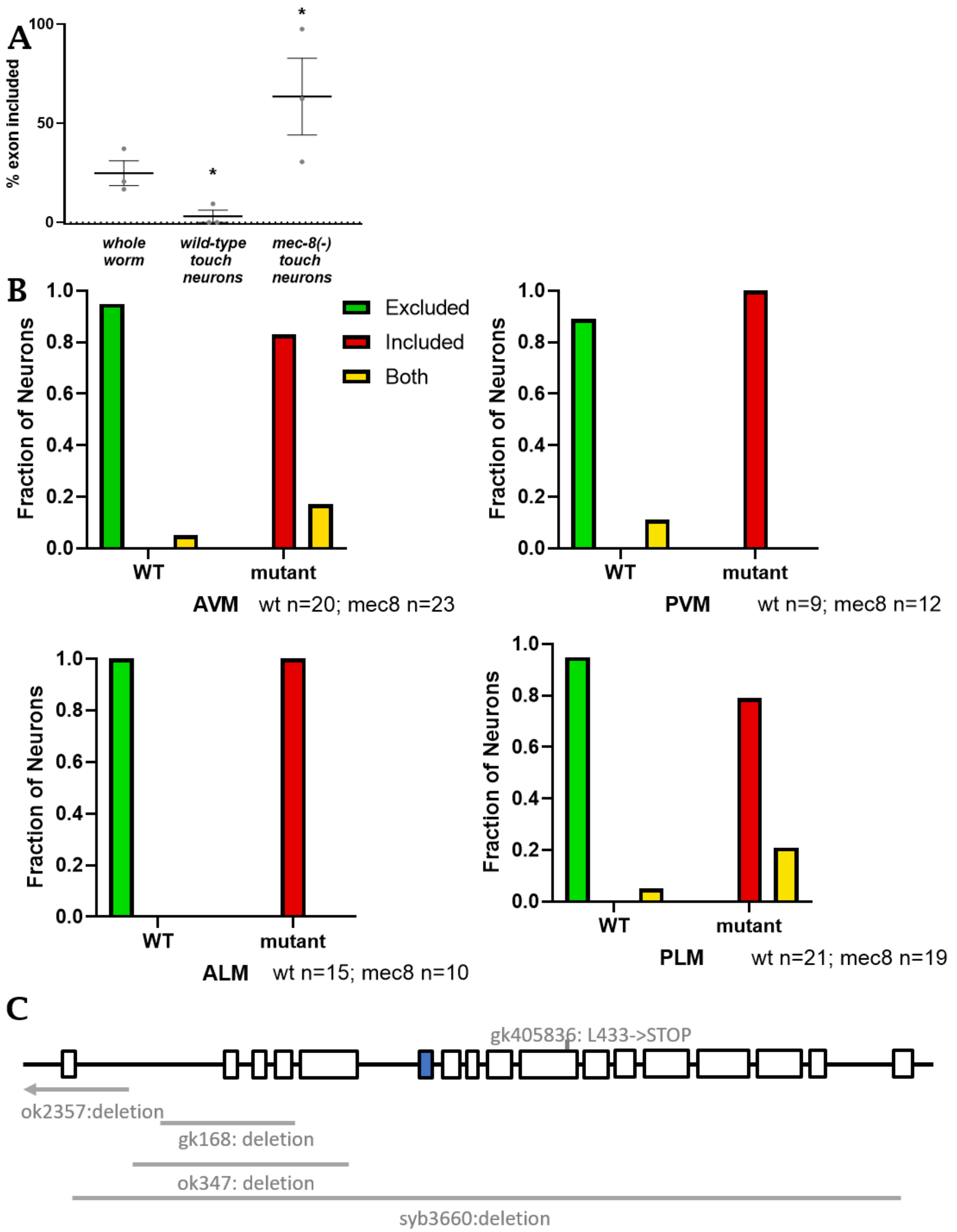

Figure S3: *elp-1* splicing is controlled by MEC-8 in touch neurons. (A) PSI for novel alternatively-spliced cassette exon in *elp-1* across biological replicates. Asterisk indicates $p<0.05$, one-way ANOVA. (B) Quantification of splicing patterns displayed in Fig 3D-F. Splicing of *elp-1* is essentially binary- nearly all wild-type worms express only the skipped isoform in all touch neurons, while nearly all *mec-8* mutant worms express only the included isoform in all touch neurons. n refers to number of different animals scored. (C) Schematic of loss-of-function *elp-1* alleles used and generated for Fig 3G.

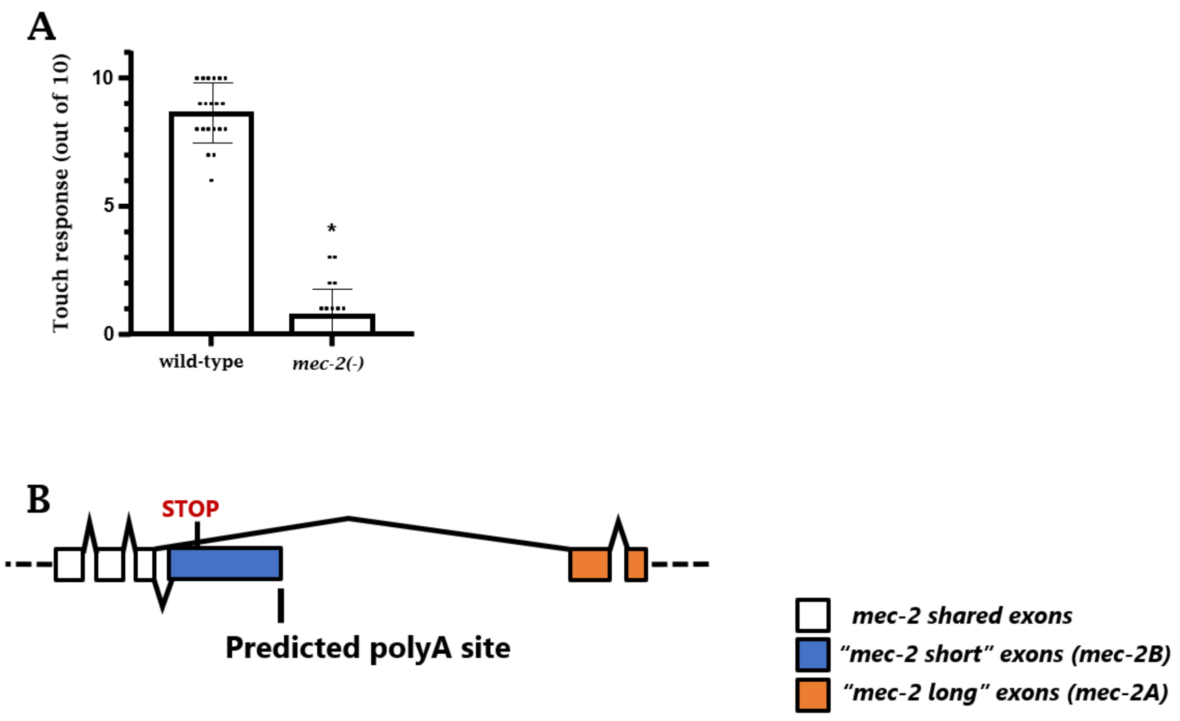

Figure S4: *mec-2* splicing and function (A) *mec-2* mutants are defective in touch sensation. Reference mutant allele *mec-2(e75)* was used. Asterisk indicates $p<0.05$, unpaired two-tailed t-test. (B) Predicted polyA site for the short *mec-2b* isoform as predicted by PolyASite 2.0.

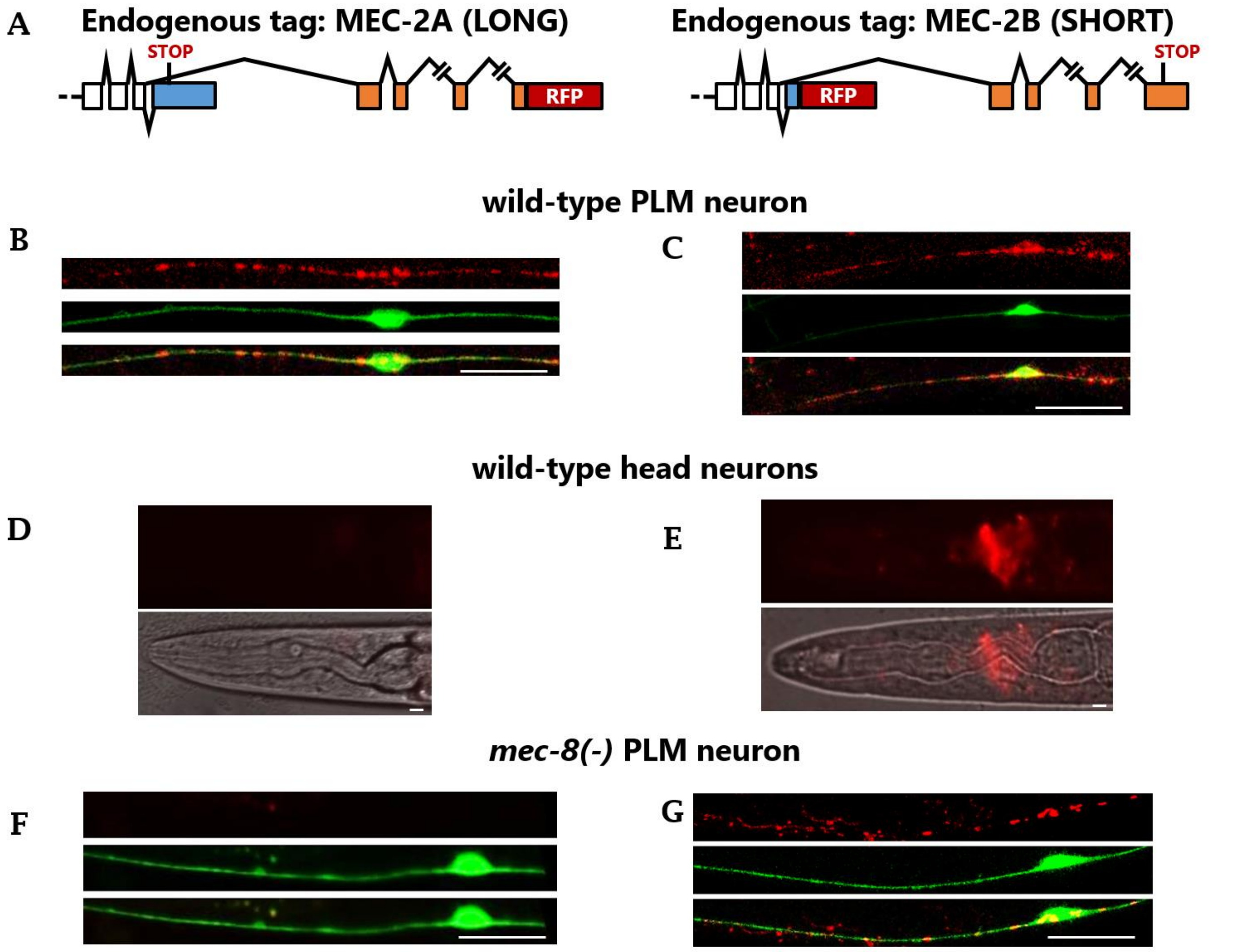

Figure S5: Endogenous *mec-2* isoforms are differentially expressed in different neuron types, and are differentially affected by *mec-8* mutation. (A) Two complementary endogenous strains were created using CRISPR/Cas9 genome editing: an RFP-tagged long *mec-2A* strain and an RFP-tagged short *mec-2B* strain. (B) MEC-2A::RFP is expressed in PLM touch neuron in a punctate pattern throughout both cell body and neurite. (C) MEC-2B::RFP is also expressed in PLM in punctate pattern throughout cell body and neurite. (D) MEC-2A::RFP is not strongly expressed in wild-type head neurons, where MEC-2B::RFP (E) is strongly expressed. (F) *mec-8* mutation results in loss of MEC-2A::RFP expression in PLM neurons. (G) *mec-8* mutation does not result in loss of MEC-2B::RFP expression in PLM neurons. Scale bars represent 5 µm.

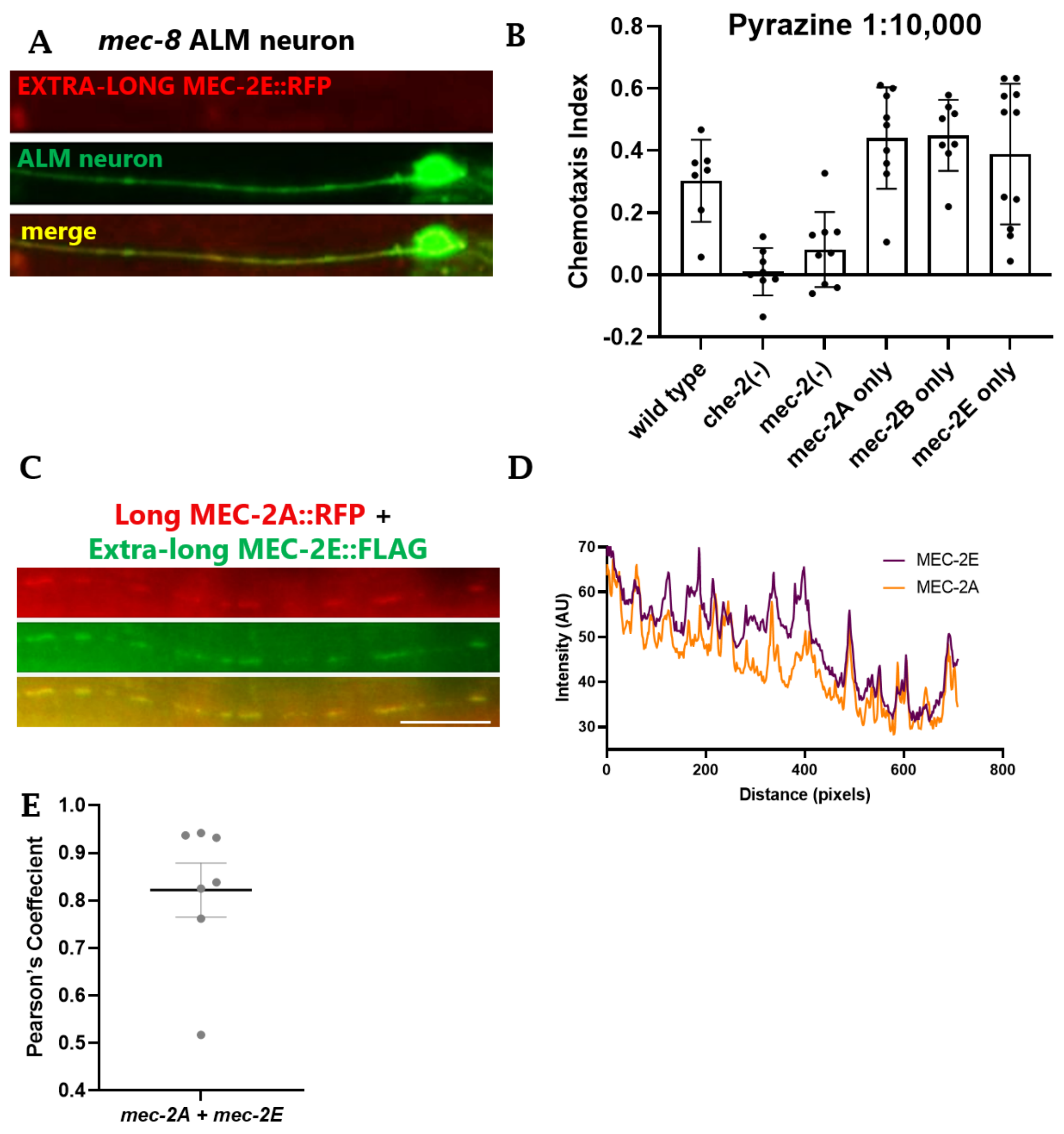

Figure S6: *mec-2E* isoform-specific expression and function. (A) MEC-2E::RFP expression in touch neurons is abolished in *mec-8* mutants (B) Forcing expression of either MEC-2A, MEC-2B, or MEC-2E does not cause strong defects in olfaction. (C) Endogenously-tagged long MEC-2A::RFP and endogenously-tagged (also via CRISPR/Cas9) extra-long MEC-2E::FLAG exhibit extensive punctate co-localization in individual neurites of touch neurons, as assessed by immunofluorescence. (D) Line scan of MEC-2A::RFP MEC-2E::FLAG image from panel C, demonstrating extensive overlap between MEC-2A::RFP and MEC-2E::FLAG puncta. (E) Quantification of Pearson's coefficient for colocalization between MEC-2A and MEC-2E fluorescence channels.

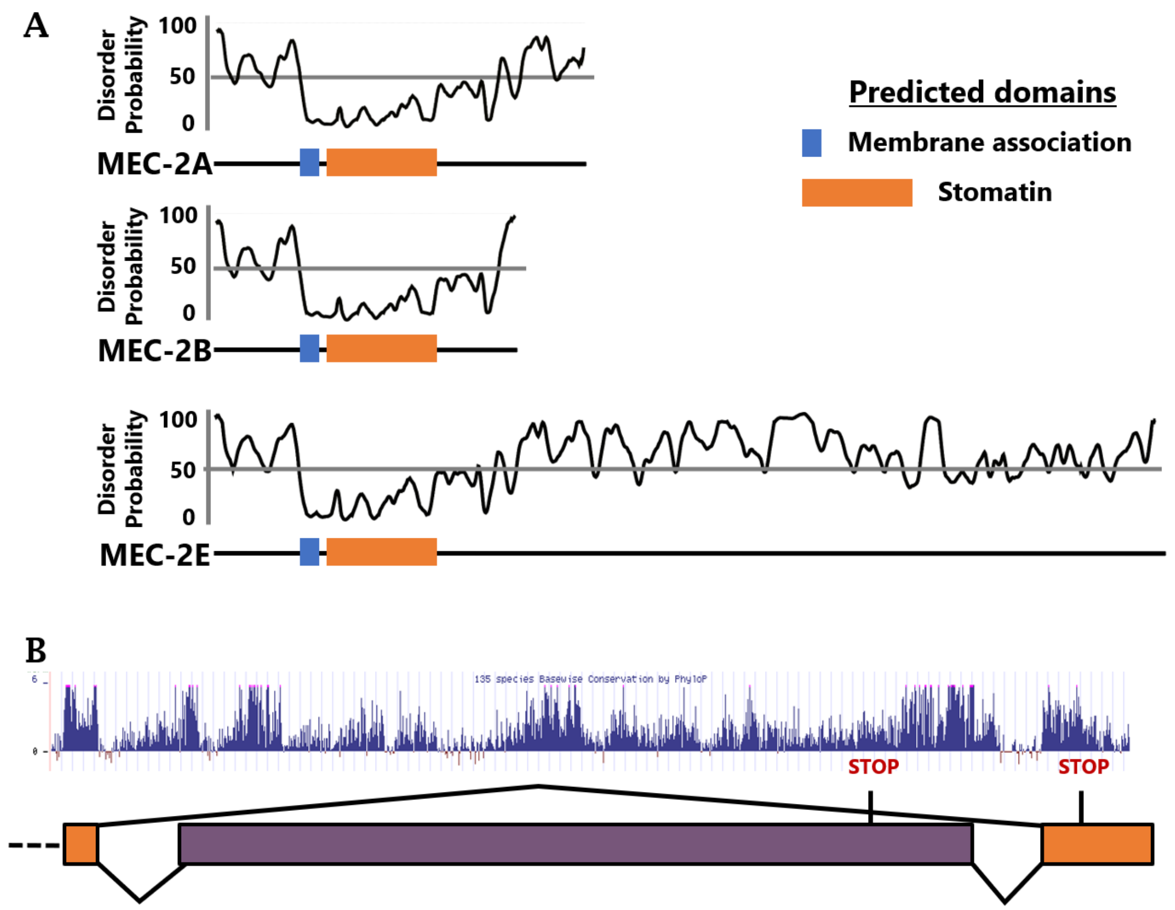

Figure S7: *mec-2* isoform-specific structure and conservation (A) Alternative C-termini of MEC-2 isoforms have no discernible protein domains, but differ in the length of their predicted regions of C-terminal intrinsic disorder. (B) Large non-canonical MEC-2E cassette exon exhibits substantial conservation among nematode species (PhyloP 135 species).
